# Mechanisms of Heshouwuyin in regulating apoptosis of testicular cells in aging rats through mitochondrial pathway

**DOI:** 10.1186/s12906-016-1323-6

**Published:** 2016-09-01

**Authors:** Jingbo Chen, Yujuan Wang, Chenhong Hui, Yao Xi, Xiang Liu, Feng Qi, Haokun Liu, Zhenshan Wang, Siyun Niu

**Affiliations:** 1 School of Medicine, Hebei University, Baoding, 071002 Hebei Province China; 2 College of Life Science, Hebei University, Baoding, 071002 Hebei Province China; 3 Baoding NO.1 Hospital Of TCM, NO.530 Yuhua West Road, Baoding, 071000 Hebei Province China; 4 Military Transportation University, NO.1 Dongjuzi, Chenglin Road, Hedong District Tianjin, 300161 China

**Keywords:** Heshouwuyin, Cell apoptosis, Mitochondrial apoptosis pathway

## Abstract

**Background:**

*Polygonum multiflorum* has important effects on anti-aging and immunity enhancement. Many traditional Chinese medicine preparations based on *Polygonum multiflorum* are widely used for the clinical prevention and treatment of aging. However the mechanisms of these herb mixtures are often unknown. This study investigates the effect of Heshouwuyin, a Chinese herbal compound for invigorating the kidney, on the regulation of testicular cells apoptosis in aging rats.

**Methods:**

In this study, 18-month-old Wistar rats served as a model of natural aging and 12-month-old rats served as a young control group. Heshouwuyin group 1 and group 2 were comprised 18-month-old rats given Heshouwuyin intragastrically for 60 days and 30 days respectively. Then testes of the young control group were isolated in the age of 12 months, the other three groups were in the age of 18 months.

**Results:**

TUNEL assay showed that the rate of testicular cell apoptosis was obviously higher and Flow cytometry analysis showed that the rate of cell proliferation was significantly lower in the natural aging group than in the young control group and that intervention with Heshouwuyin could reverse this phenomenon. Therefore, we further applied microarray analysis to screen out differentially expressed genes regulated by Heshouwuyin and related to cell apoptosis. The expression of these genes was observed by quantitative fluorescence PCR, immunofluorescence staining, and western blot. The results showed that the expression of 14-3-3σ was significantly lower and that the expression of DR6, BAX, caspase-3 and Cytc were significantly higher in the natural aging group than in the young control group, but intervention with Heshouwuyin significantly reversed this phenomenon. Moreover, the curative efficacy of Heshouwuyin after 60 days was better than that of Heshouwuyin after 30 days.

**Conclusion:**

Our study suggests that Heshouwuyin has anti-aging effects on the testis by means of inhibiting the occurrence of apoptosis in spermatogenic cells, thus improving the spermatogenic function of the testis. This is mainly achieved by regulating the expression of key genes in the mitochondrial apoptosis pathway.

## Background

*Polygonum multiflorum*, considered as one of Chinese four great panaceas, was first recorded in *Kai Yuan Ben Cao* (English title, *Kaiyuan Medical*), a book on Chinese herbal medicine. Prepared *Polygonum multiflorum* is beneficial to the liver and kidney as well as the human physique; it is able to strengthen the muscles and bones, and blacken the hair [1]. Recent studies have confirmed that *Polygonum multiflorum* improves immunity, lowers the blood fat concentration, and has obvious anti-aging effects such as anti-atherosclerosis and neuroprotective effects [2, 3]. Additionally, it is associated with little toxicity and few side effects. Many traditional Chinese medicine preparations based on *Polygonum multiflorum* are widely used for the clinical prevention and treatment of aging; such preparations include Heshouwu pills, Qidanbaomeisong pills and Shouwu yanshou. Previous studies have shown that Heshouwuyin up-regulates the level of serum testosterone and down-regulates the expression of Cox7a2 in testis tissue of exercised-induced fatigue rats [4], moreover, Heshouwuyin regulates hypothalamic-pituitary-testicular secretion of gonadotropin-releasing hormone, gonadotropin and insulin-like growth factor-1 [5]. Recent studies have found that Heshouwuyin improves the expression of testosterone synthesis enzyme in testicular Leydig cells, promotes the secretion of testosterone, and improves the sperm quality of natural aging rats [6]. Heshouwuyin may also up-regulate Bcl-2 protein and down-regulate Bax protein in the testicular Leydig cells of over-training rats as well as reduce to damage Leydig cells [7]. However, the mechanism by which Heshouwuyin regulates the apoptosis of testicular cells in aging rats remains unclear. In this study, microarray analysis technology was used to screen out differentially expressed genes that are associated with apoptosis and regulated by Heshouwuyin. Next, quantitative real-time polymerase chain reaction (qRT-PCR), immunofluorescence, and western blot were used to observe the expression of several genes in the mitochondrial apoptosis pathway. The purpose of this study was to further explore the mechanisms of Heshouwuyin in delaying testes aging and regulating spermatogenesis.

## Methods

### Design

This study was a randomized controlled animal experiment.

### Time and setting

The experiment was completed at the School of Life Science, Hebei University, from April 2013 to March 2014.

### Materials

Fifty five clean-grade male Wistar rats weighing 350 to 390 g were provided by the Experimental Animal Laboratory, Quality Inspection Center of Shandong Lukang Pharmaceutical Group Co., Ltd., P.R. China (license No. 20080001). Disposal of experimental animals was performed in accordance with the *Guidance Suggestions for the Care and Use of Laboratory Animals*, formulated by the Ministry of Science and Technology of China. The experimental procedures were conducted according to the guidelines by the Animal Care and Ethics Committee of Hebei University, P.R. China.

### Chinese herbal compound preparation

#### Heshouwuyin prescription

The Heshouwuyin prescription used in this study comprised *Polygonum multiflorum*, *Cistanche deserticola*, *Radix Achyranthis Bidentatae*, *Epimedium* spp., *Salvia miltiorrhiza*, and *Poria cocos*. All herbs were purchased from the Hebei Hospital of Traditional Chinese Medicine. The herbs were cut into pieces and mixed in a mass ratio of 3:2:3:2:5:3, respectively. The mixture was immersed in distilled water that was eight times the mass of the mixture for 1 h, decocted with water twice (once for 30 min), and then filtered and concentrated. The mixture (final concentration of 4.8 g/mL) was stored at 4 °C until use. The herbal compound was rewarmed to 25 to 30 °C before administration.

#### Drug dose

According to the adult dose conversion, 100 g of decocted Heshouwuyin containing 2.4 g of crude drug is equivalent to the adult dose. Our preliminary findings implicated that twice the adult dose produced the best effects. Therefore, 100 g of Heshouwuyin containing 4.8 g of crude drug was considered the administration dose in the present study. Heshouwuyin group 1 was intragastrically administered a dose of 4.8 g/100 g body weight for 60 days and Heshouwuyin group 2 was intragastrically administered a dose of 4.8 g/100 g body weight for 30 days.

### β-galactosidase enzyme assay

A β-galactosidase staining kit was used (GMS10012.3; Genmed Scientific Inc., Wilmington, DE, USA). According to the experimental requirements, 8-μm-thick sections of testis tissue were washed in β-galactosidase cleansing solution for 5 min and fixed using β-galactosidase fixative for 10 min. The fixative was then aspirated, and the sections were treated with acid solution three times for 5 min. The sections were incubated with β-galactosidase dye working solution (19:1 dilution:staining solutions) at 37 °C overnight, then washed in β-galactosidase cleansing solution for 5 min. Under an optical microscope (Olympus E53; Olympus, Tokyo, Japan), the cytoplasm of β-galactosidase-positive cells was blue. The number of blue cells among 500 cells was observed and recorded. The experiments were repeated three times for robust statistical analysis.

### In situ germ cell apoptosis detection and quantification

To determine the percentage of apoptotic cells in each sample, we performed terminal deoxynucleotidyl transferase-mediated dUTP nick-end labeling assay (TUNEL) staining using the DeadEnd™ Fluorometric TUNEL System (Promega, Madison, WI, USA). First, 8-μm-thick frozen sections of testis tissue were washed three times in phosphate-buffered saline (PBS) for 10 min. The slides were then permeabilized with 0.5 % Triton X-100 in 0.1 % sodium citrate for 20 min. The permeabilized sections were washed in PBS, pre-balanced by buffer for 10 min, and then covered with TUNEL reaction mixture in a dark room at 37 °C for 1 h. The sections were treated with termination by 2 × SCC converter at room temperature for 15 min. After four washes in PBS, the sections were incubated with 4′,6-diamidino-2-phenylindole (DAPI) for 20 min at room temperature, washed, and mounted in a fluorescence protector medium. The sections were evaluated using fluorescence microscopy. The number of green cells among 500 cells was observed and recorded. Three samples from each group were analyzed.

### Flow cytometry to detect amount of DNA in testicular cells

The appropriate amount of frozen sections from the testis tissue was rewarmed at 42 °C, the white film and fat pad were removed, and the sections were placed in a petri dish. Next, 1 ml of 0.01 M precooling PBS (pH 7.2–7.4) was added to the petri dish, the sections were cut into pieces, and the suspension was placed into the loading slot of a tissue sample preparation instrument (BD Medimachine, TY4123; BD Biosciences, Franklin Lakes, NJ, USA) and broken for 1 min. The cell suspension was suctioned out and centrifuged at 1500 × *g* for 10 min (Eppendorf 5424 Microcentrifuge; Fisher Scientific, Waltham, MA, USA), and the supernatant was discarded. Next, 1 ml of 70 % precooling ethanol was added to the precipitation, and the sample was pipetted up and down and stored at 4 °C overnight. The cell suspension was removed and centrifuged at 1500 × *g* for 8 min, and the ethanol supernatant was discarded. The suspension was resuspended in 1 ml of PBS, centrifuged at 1500 × *g* for 8 min, and resuspended; this was repeated twice. Next, 500 μl of propidium iodide was added to the precipitation, incubated for 30 min at 4 °C, and filtered with a 200-mesh sieve. Flow cytometry was performed using a FACS420 (Becton Dickinson, San Jose, CA, USA). For quantitative analysis, data were input into a Consort-30 computer for processing. The proliferation index, which indicates the cell proliferation activity, was calculated as follows:

$$ \mathrm{PI}\left(\%\right) = \frac{S+G2/M}{G0/G1+S+G2/M} \times 100\% $$

### Gene microarray hybridization and data analysis

Randomly selected testis tissue from rats of the young control group, natural aging group, and Heshouwuyin group 1 were placed into the RNAlater, and gene microarray hybridization was carried out by Shanghai Biotechnology Corporation (SBC). After obtaining the original data, the fluorescence signal intensity was normalized by the cubic spline method, and the SBC online system provided by the company (http://www.shbiochip.bioon.com.cn/) was applied. Differentially expressed genes were selected by the fold-change method (FC method), in which the principle of choosing differential genes is FC ≥ 2.0 or FC ≤ 0.5.

### RNA isolation and qRT-PCR

To verify the accuracy of microarray data, differentially expressed genes were chosen to quantify their mRNA expression levels by qRT-PCR. First, we prepared total RNA from testis tissue using Trizol reagent (Invitrogen, Carlsbad, CA, USA) according to the manufacturer’s instructions. After reverse transcription, the resulting materials were used for qRT-PCR amplification using gene-specific primer pairs (Table 1) and SYBR Green PCR Master Mix (Applied Biosystems, Foster City, CA, USA).

**Table 1:** Primer sequences and annealing temperature of each gene (Table 1 should be listed at the end of 2.9 RNA isolation and qRT-PCR, on the Page 8)

Gene	Primer sequences	Annealing	Product
		Tm (°C)	size (bp)
Ccng1	F:CCTTCCAATTTCTGCAGCTC	60 °C	281
	R:CTTGGAAACAAGCTCTTGCC		
GHR	F:ATCTTTGGCGGGTGTTCTTA	60 °C	78
	R:TGTTGGCTATCTCGTAGTGGA		
Cabin1	F:AGTCCAGCAGAGCCAAGTCC	60 °C	313
	R:TGAACCCGTCATACGTCCAT		
Capn8	F:ACGCTGTCTACCAGATTCCC	64 °C	342
	R:TGCCCACAAACTCCTCAAAC		
AK1	F:GTGGACGATAACGAGGAG	60 °C	166
	R:TCAGGGAGTCAAGATAGGTG		
Cybrd1	F:CTTCGTACCATTCATTCCCACC	58 °C	171
	R:CCATTCCGTCTGCGTTGC		
Kdm2b	F:TGCCGAGATGAAATACCC	60 °C	170
	R:CATACAGAGCCAAGTTGTGC		
Glrx3	F:AGCACCCAAGTTAGAGGA	60 °C	292
	R:TAGCAATTCACCGTTGTC		
ADAM5	F:CCGTTGAAATCTGGTCG	55 °C	107
	R:AATGTGCTGCGGTCTAT		
BAX	F:GCGATGAACTGGACAACAACAT	62 °C	153
	R:TAGCAAAGTAGAAAAGGGCAACC		
caspase-3	F:GACTGCGGTATTGAGACAGA	60 °C	209
	R:CGAGTGAGGATGTGCATGAA		
DR6	F:CAGACCATGAACGAGCCT	60 °C	142
	R:GTATCTTCCATCAGCCCAC		
14-3-3σ	R:TAGCTGGTGTAGCCCCACTT	55 °C	95
	F:CATGGACATCAGCAAGAAGGA		
GSY2	F:CCTCGATGGCTGTGATTTCTGACAC	60 °C	172
	R:CTTGGGCGTTATCTCTGTGCAGCAA		
SOCS3	F:CTGGACCCATTCGGGAGTTC	62 °C	105
	R:AACTGGGAGCTACCGACCATTG		
β-actin	F:GACGTTGACATCCGTAAAGACC	60 °C	115
	R:TGCTAGGAGCCAGGGCAGTA		

### Immunofluorescence

Frozen 8-μm-thick sections of testis tissue were washed twice in PBS for 10 min. The slides were then permeabilized with 0.5 % Triton X-100 for 10 min. Nonspecific sites were blocked by incubation with 5 % serum albumin for 3 h at room temperature before incubating the section with the following primary antibodies: BAX, DR6, caspase-3, 14-3-3σ and Cytc (BAX, DR6, 14-3-3σ, 1:100; Santa Cruz Biotechnology, Santa Cruz, CA, USA; caspase-3, 1:100; Cell Signaling Technology, Beverly, MA, USA; Cytc, 1:100; Abcam, Cambridge, UK) at 4 °C overnight. The next day, the sections were washed with PBS and incubated for 1 h at 37 °C with secondary antibodies. After four washes in PBS, the sections were incubated with DAPI for 20 min at room temperature, washed, and mounted in a fluorescence protector medium (Electron Microscopy Sciences, Hatfield, PA, USA). The number of positive cells among 500 cells was observed and recorded by confocal microscopy.

### Western blotting

Western blotting was performed using testicular lysates. Cytoplasmic proteins were isolated from the testes according to the protocols of the Tissue Mitochondria Isolation Kit (Beyotime Institute of Biotechnology, Jiangsu, China). The protein concentration was quantified using a bicinchoninic acid protein assay kit (Pierce BCA Protein Assay Kit; Thermo Scientific, Waltham, MA, USA). In brief, protein extracts from each sample were added to a 5× gel loading buffer and boiled for 3 min. Equal amounts of protein (100 μg) were separated by SDS-PAGE and transferred onto PVDE membranes. The membranes were blocked in 5 % nonfat powdered milk in Tris-buffered saline for 1 h. The membranes were then incubated overnight at 4 °C with the appropriate primary antibodies (BAX, DR6, 14-3-3σ, 1:200, Santa Cruz Biotechnology; caspase-3, 1:400; Cell Signaling Technology; Cytc, 1:400; Abcam). After three washes in Tris-buffered saline/Tween 20 for 10 min each, the membranes were incubated for 1 h in the dark with the appropriate IRDye 800-conjugated secondary antibodies (1:1,0000; LI-COR Biosciences, Inc. Lincoln, NE, USA). The signals were detected using the Odyssey Imaging System (LI-COR Biosciences, Inc.).

### Statistical analysis

Data are expressed as mean ± standard deviation (SD) and were analyzed using SPSS 16.0 statistical software (SPSS, Inc., Chicago, IL, USA). Normal distribution was assessed by Q–Q plots and variance homogeneity by robust variance tests. The data showed a normal distribution and homogeneity of variance. Differences between groups were compared using one-way analysis of variance. Intergroup comparison was performed using the Student–Newman–Keuls q test. A value of *P* < 0.05 was considered statistically significant.

## Results

### Animals and tissue collection

#### Animals

Fifty five 12-month-old male Wistar rats were randomly divided into four groups after 1 week of adaptive feeding. The young control group (YCG) (*n* = 10, 12 months of age) and the natural aging group (NAG) (*n* = 15, 16 months of age) were both intragastrically administered normal saline for 60 days. Rats in the Heshouwuyin group 1 (SWY1G) (*n* = 15, 16 months of age) were given Heshouwuyin (4.8 g/100 g body weight) intragastrically for 60 days. Rats in the Heshouwuyin group 2 (SWY2G) (*n* = 15, 17 months of age) were given Heshouwuyin (4.8 g/100 g body weight) intragastrically for 30 days. After exclusion of rats with cancer or other diseases, 10 rats in each group were included in this study.

### Collection of testicular tissues

After weighing, five rats in each group were anesthetized using 6 % chloral hydrate (0.5 mL/100 g body weight). With 75 % ethanol disinfection, the abdominal cavity was opened to remove the bilateral testicular tissues quickly. Randomly selected testicular tissues from rats of the YCG, NAG, and SWY1G were placed into the RNAlater for gene microarray hybridization, the rest of the testicular tissues were put into liquid nitrogen for 5 min, and then were transferred to −80 °C for later use; the other rats in each group were anesthetized using 6 % chloral hydrate and were perfused using 4 % paraformaldehyde. After 75 % ethanol disinfection, the abdominal cavity was opened, and the bilateral testicular tissues were quickly removed, tissues were fixed in 4 % paraformaldehyde and dehydrated in 30 % sucrose solutions. The fixed tissues were prepared for immunofluorescence.

### Expression of β-galactosidase in different experimental groups’ testes

**Fig. 1:**
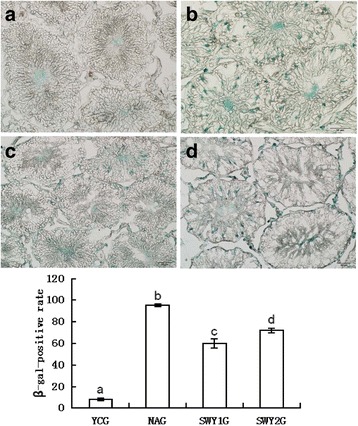
β-Galactosidase staining and statistical analysis in different experimental groups. Cells positive for β-galactosidase stained blue. The β-galactosidase in all groups was distributed among the Leydig cells; in the NAG, the positive cells were also located among the spermatogonia. Statistical analysis showed that the β-galactosidase-positive rate was significantly higher in the NAG than YCG (*P* < 0.01), while the SWY1G and SWY2G showed a significantly lower rate (*P* < 0.01). **a** young control group (YCG); **b**: natural aging group (NAG); **c**: Heshouwuyin group 1 (SWY1G); **d**: Heshouwuyin group 2 (SWY2G). Each histogram represents the mean ± SD (*n* = 3). Columns with different letters represent significantly different values, while the same letters indicate no significant difference. Bar = 50 μm

β-Galactosidase is located in the cytoplasm, and cells positive for β-galactosidase stained blue. There were few positive cells in the YCG, and the positive staining was mainly distributed in the Leydig cells; the number of positive cells was significantly higher in the NAG than in the YCG, and these cells were mainly distributed in the spermatogonia and Leydig cells. The β-galactosidase-positive rate in the NAG was significantly higher than that in the YCG (*P* < 0.01). After Heshouwuyin intervention, the positive cells were distributed among the Leydig cells and the rate of positive cells was significantly lower than that in the NAG (*P* < 0.01),and the rate of positive cells in the SWY1G was significantly lower than that in the SWY2G (*P* < 0.01) (Fig. 1).

### Apoptosis of testicular cells in different experimental groups

**Fig. 2:**
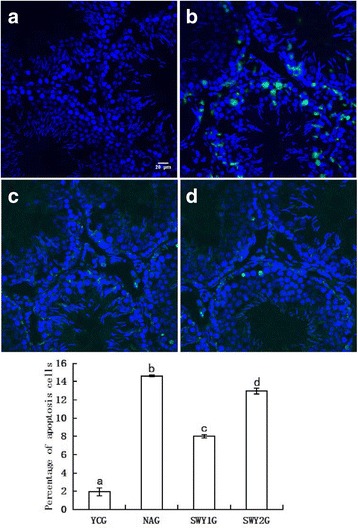
TUNEL staining showing apoptosis of testicular cells and statistical analysis in different experimental groups. TUNEL staining indicated that the nucleus of apoptotic cells was green. In the YCG, positive staining was found only in Leydig cells; in the NAG, positive staining was found in spermatogonia, spermatocytes, sperm cells, and Leydig cells; and in the SWY1G and SWY2G, positive staining were found in spermatogonia and Leydig cells. Statistical analysis showed that the rate of apoptosis was significantly higher in the NAG than in the YCG (*P* < 0.01) and significantly lower in the SWY1G and SWY2G than in the NAG (*P* < 0.01). **a**: young control group (YCG); **b**: natural aging group (NAG); **c**: Heshouwuyin group 1 (SWY1G); **d**: Heshouwuyin group 2 (SWY2G). Each histogram represents the mean ± SD (*n* = 3). Columns with different letters represent significantly different values, while the same letters indicate no significant difference. Bar = 20 μm

The apoptosis of testicular cells in each group was observed by TUNEL staining. The nucleus of positive cells was green, while normal nuclei appeared blue as stained by DAPI. Laser confocal microscopy showed few apoptotic cells in the YCG and occasional positive cells among the Leydig cells. In the NAG, there was a large number of apoptotic cells in the seminiferous tubules; spermatogonia, spermatocytes, sperm cells, and Leydig cells were occasionally apoptotic. In the SWY1G and SWY2G, the number of apoptotic cells was significantly lower than in the NAG, and the apoptotic cells were mainly distributed among the spermatogonia and Leydig cells. Statistical analysis showed that the rate of apoptosis was significantly higher in the NAG than in the YCG (*P* < 0.01) and significantly lower in the SWY1G and SWY2G than in the NAG (*P* < 0.01) and the rate of apoptosis in SWY1G was significantly lower than in the SWY2G (*P* < 0.01) (Fig. 2).

### Cell cycles of testicular spermatogenic cells in different experimental groups detected by flow cytometry

**Fig. 3:**
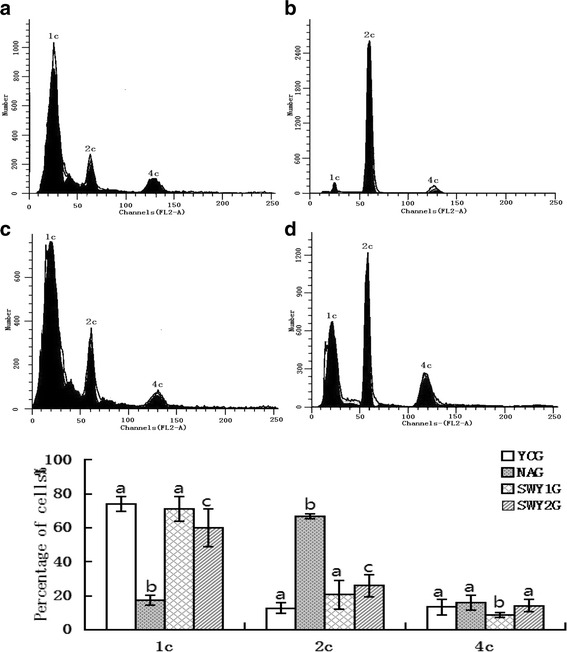
Cell cycles in different experimental groups detected by flow cytometry. Cell cycles were detected by flow cytometry, and the results are indicated by the haploid number. Cells in the YCG were mainly haploid; cells in the NAG were mainly diploid, while the haploid cells were significantly decreased (*P* < 0.05); and the rate of haploid cells was significantly higher in the SWY1G and SWY2G than in the NAG (*P* < 0.05).1c: haploid; 2c: diploid; 4c: tetraploid. **a**: young control group (YCG); **b**: natural aging group (NAG); **c**: Heshouwuyin group 1 (SWY1G); **d**: Heshouwuyin group 2 (SWY2G). Each histogram represents the mean ± SD (*n* = 5). Columns with different letters represent significantly different values, while the same letters indicate no significant difference

**Fig. 4:**
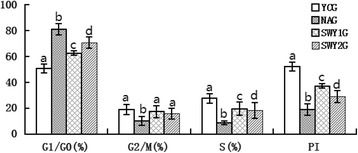
Cell cycles phase in different experimental groups detected by flow cytometry. Cell cycle phases in the different experimental groups were detected by flow cytometry. Statistical analysis showed that the rate of cells in the G1/G0 phase was significantly higher and that the rate of cells in the G2/M and S phases was significantly lower in the NAG than in the YCG (*P* < 0.05). The rate of cells in the G1/G0 phase was significantly lower and the rate of cells in the G_2_/M and S phases was significantly higher in the SWY1G and SWY2G than in the NAG (*P* < 0.05). YCG: young control group; NAG: natural aging group; SWY1G: Heshouwuyin group 1; SWY2G: Heshouwuyin group 2. PI, proliferation index. Each histogram represents the mean ± SD (*n* = 5). Columns with different letters represent significantly different values, while the same letters indicate no significant difference

The cell cycles of testicular spermatogenic cells in the different experimental groups were detected by flow cytometry. The spermatogenic cells in the YCG were mainly haploid; the numbers of haploid were significantly lower in the NAG than YCG, and the spermatogenic cells were mainly diploid. The rate of haploid was significantly higher in the SWY1G and SWY2G than in the NAG (*P* < 0.05) (Fig. 3). The number of cells in G1/G0 cycle arrest was significantly higher in the NAG than in the YCG (*P* < 0.05), and the cell proliferation index was significantly lower in the NAG than in the YCG (*P* < 0.05). Heshouwuyin administration improved this phenomenon; the cell proliferation index was higher and the number of cells in G1/G0 cycle arrest was lower in the SWY1G and SWY2G than in the NAG (*P* < 0.05), the number of cells in G1/G0 cycle arrest was lower and the cell proliferation index was higher in the SWY1G than in the SWY2G (Fig. 4).

### Gene microarray analysis results

#### Differential expression genes screened out by gene microarray analysis

**Fig. 5:**
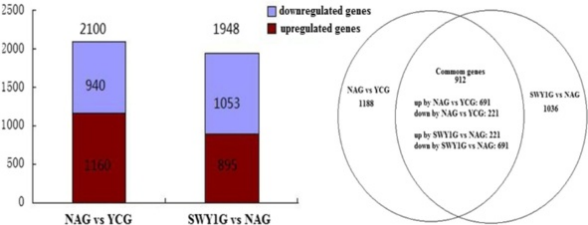
Differentially expressed genes regulated by Heshouwuyin

Agilent single standard gene expression profile chip was detected by SBC. In total, 21,260 genes were screened out, 2100 genes were differentially expressed between the YCG and NAG, 1948 genes were differentially expressed between the SWY1G and NAG, and 912 genes were directly regulated by Heshouwuyin. Of the latter 912 genes, 691 were significantly up-regulated in the NAG and 221 were significantly down-regulated compared with the YCG. After Heshouwuyin intervention, the 691 genes were significantly down-regulated, the 221 genes were significantly up-regulated, and 65 of the 912 genes were related to cell apoptosis (Fig. 5).

#### Gene ontology analysis

Gene ontology analysis showed that genes regulated by Heshouwuyin were mainly involved in reproduction, immune function, metabolism, cell growth and development, death and a variety of other biological processes. Additionally, these genes participated in energy metabolism, catalytic processes, signal transduction, molecular synthesis, folding, circulation, transportation and nutrition.

#### Pathway analysis

Pathway analysis showed that the 912 differentially expressed genes regulated by Heshouwuyin were mainly distributed in 59 pathways, 12 of which were cell cycle regulation-related and apoptosis-related pathways (Table 2).

**Table 2:** Twelve pathways related to cell cycle regulation and apoptosis, including genes directly regulated by Heshouwuyin

Category	Number of related genes
Jak-STAT signaling pathway	9
p53 signaling pathway	6
Wnt signaling pathway	8
MAPK signaling pathway	10
IGF-1 signaling pathway	2
ErbB signaling pathway	4
mTOR signaling pathway	3
Calcium signaling pathway	8
Hedgehog signaling pathway	3
Regulation of BAD phosphorylation	2
ERK1/2 Mapk signaling pathway	3
Role of MEF2D in T-cell Apoptosis	2

#### Confirmation of typical gene expression from gene microarray data by qRT-PCR

**Fig. 6:**
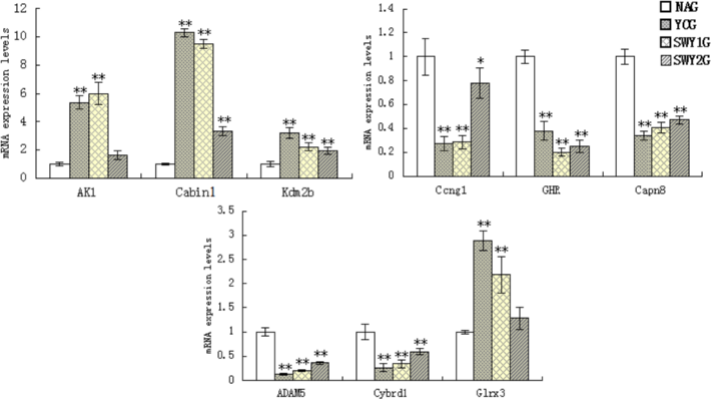
qRT-PCR for nine typical genes. qRT-PCR was used to confirm the accuracy of the microarray results. Nine typical genes were detected. Compared with the YCG, four genes (AK1, Glrx3, Cabin1 and Kdm2b) were down-regulated and five genes (Ccng1, GHR, Capn8, ADAM5 and Cybrd1) were up-regulated in the NAG. The Heshouwuyin intervention reversed these trend. YCG: young control group; NAG: natural aging group; SWY1G: Heshouwuyin group 1; SWY2G: Heshouwuyin group 2. Each histogram represents the mean ± SD (*n* = 3). *P < 0.05, **P < 0.01

To confirm the accuracy of the microarray results, we detected the expression pattern of nine typical genes (AK1, Cabin1, Kdm2b, Ccng1, GHR, Capn8, ADAM5, Cybrd1 and Glrx3) using qRT-PCR (Fig. 6). Based on the qRT-PCR data, all nine genes showed differential expression in the NAG versus YCG and in the SWY1G, SWY2G versus NAG, although the variation was not as dramatic as shown by the microarray data. The qRT-PCR results were generally consistent with those of the microarray analysis (Table 3).

**Table 3:** Validation results of microarray analysis by qRT-PCR

Gene ID	Gene symbol	Fold change (NAG vs YCG)		Fold change (SWY1G vs NAG)		Fold change (SWY2G vs NAG)
		Microarry	qRT-PCR	Microarray	qRT-PCR	qRT-PCR
11636	AK1	0.14	0.19	3.6	6.0	1.63
30926	Glrx3	0.42	0.35	2.04	2.18	1.28
104248	Cabin1	0.47	0.1	3.24	9.52	3.34
30841	Kdm2b	0.49	0.31	2.24	2.22	1.96
12450	Ccng1	6.54	3.68	0.16	0.28	0.78
14600	GHR	6.21	2.64	0.15	0.2	0.25
170725	Capn8	6.16	2.95	0.16	0.41	0.47
11499	ADAM5	3.21	7.29	0.15	0.19	0.37
73649	Cybrd1	3.00	3.73	0.24	0.34	0.6

### qRT-PCR for key genes regulated by Heshouwuyin in the mitochondrial apoptotic pathway

**Fig. 7:**
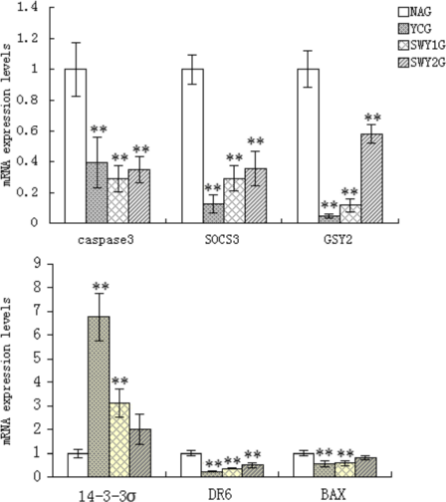
qRT-PCR for key genes regulated by Heshouwuyin in the mitochondrial apoptotic pathway. The expression levels of key genes in the mitochondrial apoptotic pathway were detected by qRT-PCR. Compared with the YCG, caspase-3, SOCS3, GSY2, BAX and DR6 were up-regulated and 14-3-3σ was down-regulated in the NAG; however, Heshouwuyin significantly reversed these trend. YCG: young control group; NAG: natural aging group; SWY1G: Heshouwuyin group 1; SWY2G: Heshouwuyin group 2. Each histogram represents the mean ± SD (*n* = 3). *P < 0.05, **P < 0.01

qRT-PCR was carried out to detect the expression levels of several key genes in the mitochondrial apoptotic pathway (caspase-3, DR6, BAX, 14-3-3σ, SOCS3 and GSY2).mRNA was collected from the testes of rats in the different groups. The results showed that compared with the YCG, the expression levels of caspase-3, DR6, BAX, SOCS3 and GSY2 were up-regulated and 14-3-3σ was down-regulated in the NAG (*P* < 0.01). In the SWY1G, caspase-3, DR6, BAX, SOCS3 and GSY2 were significantly down-regulated and 14-3-3σ was significantly up-regulated compared with the NAG (*P* < 0.01).In the SWY2G, caspase-3, DR6, SOCS3 and GSY2 were significantly down-regulated compared with the NAG (*P* < 0.01) (Fig. 7). To further confirm the location and content of these genes in the testes, immunofluorescence and western blot were subsequently carried out.

### Immunofluorescence and western blot for genes (caspase-3, DR6, BAX, 14-3-3σ and Cytc) in the mitochondrial apoptotic pathway

#### Expression of caspase-3 protein

**Fig. 8:**
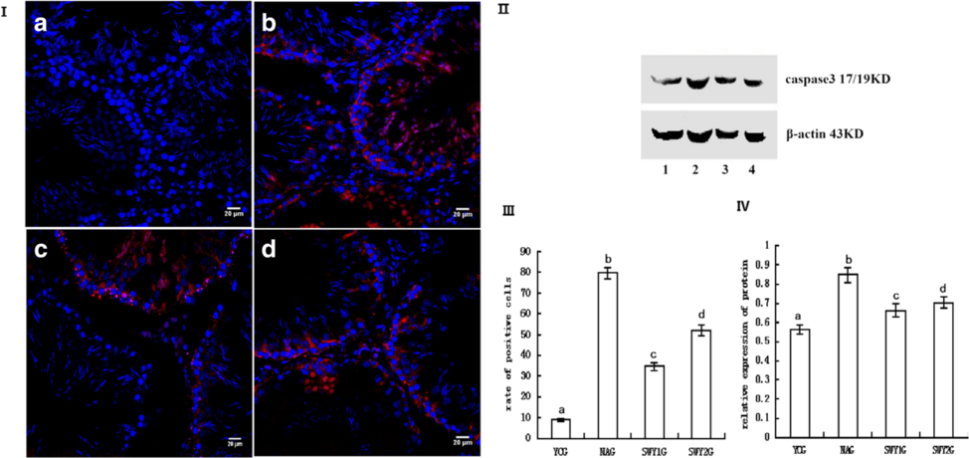
Immunofluorescence and western blot for caspase-3 and statistical analysis. Immunofluorescence staining was used to detect the expression of caspase-3 and the rates of positive cells were quantified(I,III). The results showed that the rates of cells positive for caspase-3 were significantly higher in the NAG than in the YCG (*P* < 0.01). In the SWY1G and SWY2G, the rate of cells positive for caspase-3 was significantly lower than in the NAG (*P* < 0.01). **a**: young control group (YCG); **b**: natural aging group (NAG); **c**: Heshouwuyin group 1 (SWY1G); **d**: Heshouwuyin group 2 (SWY2G). Western blot was used to detect the content of caspase-3 protein and statistical analysis was performed according to the gray level(II,IV). The results showed the variation trend was consistent with the results of immunofluorescence.1: young control group (YCG); 2: natural aging group (NAG); 3: Heshouwuyin group 1 (SWY1G); 4: Heshouwuyin group 2 (SWY2G). Each histogram represents the mean ± SD (*n* = 3). Columns with different letters represent significantly different values, while the same letters indicate no significant difference. Bars = 20 μm

Immunofluorescence staining showed that the positive products of caspase-3 were red granules distributed throughout the nucleus and cytoplasm. Normal nuclei were stained blue by DAPI. In the YCG, caspase-3 protein was mainly located in the spermatogonia of the testes; in the NAG, SWY1G and SWY2G, caspase-3 protein was mainly located in spermatogonia, spermatocytes and a few sperm cells. The rate of positive cells was significantly higher in the NAG than in the YCG (*P* < 0.01) and significantly lower in the SWY1G, SWY2G than in the NAG (*P* < 0.01), the rate of positive cells in the SWY1G was significantly lower than in the SWY2G (*P* < 0.01). Western blot was used to detect the content of caspase-3 in the testes. Semi-quantitative statistical analysis showed the variation trend was consistent with the results of immunofluorescence (Fig. 8).

### Expression of BAX protein

**Fig. 9:**
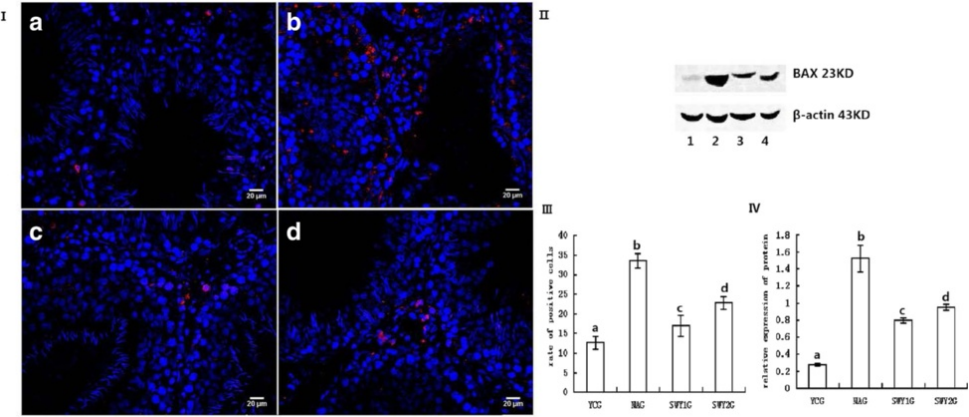
Immunofluorescence and western blot for BAX and statistical analysis. Immunofluorescence staining was used to detect the expression of BAX and the rates of positive cells were quantified(I,III). The results showed that the rates of cells positive for BAX were significantly higher in the NAG than in the YCG (*P* < 0.01). In the SWY1G and SWY2G, the rate of cells positive for BAX was significantly lower than in the NAG (*P* < 0.01). **a**: young control group (YCG); **b**: natural aging group (NAG); **c**: Heshouwuyin group 1 (SWY1G); **d**: Heshouwuyin group 2 (SWY2G). Western blot was used to detect the content of BAX protein and statistical analysis was performed according to the gray level(II,IV). The results showed the variation trend was consistent with the results of immunofluorescence.1: young control group (YCG); 2: natural aging group (NAG); 3: Heshouwuyin group 1 (SWY1G); 4: Heshouwuyin group 2 (SWY2G). Each histogram represents the mean ± SD (*n* = 3). Columns with different letters represent significantly different values, while the same letters indicate no significant difference. Bars = 20 μm

Immunofluorescence staining showed that the positive products of BAX were red granules distributed throughout the cytoplasm. In the YCG, SWY1G and SWY2G, BAX protein was mainly located in Leydig cells; in the NAG, BAX protein was mainly located in Leydig cells and a few spermatogonia. The rate of positive cells was significantly higher in the NAG than in the YCG (*P* < 0.01) and significantly lower in the SWY1G, SWY2G than in the NAG (*P* < 0.01), the rate of positive cells in the SWY1G was significantly lower than in the SWY2G (*P* < 0.01). Western blot was used to detect the content of BAX in the testes. Semi-quantitative statistical analysis showed the variation trend was consistent with the results of immunofluorescence (Fig. 9).

### Expression of DR6 protein

**Fig. 10:**
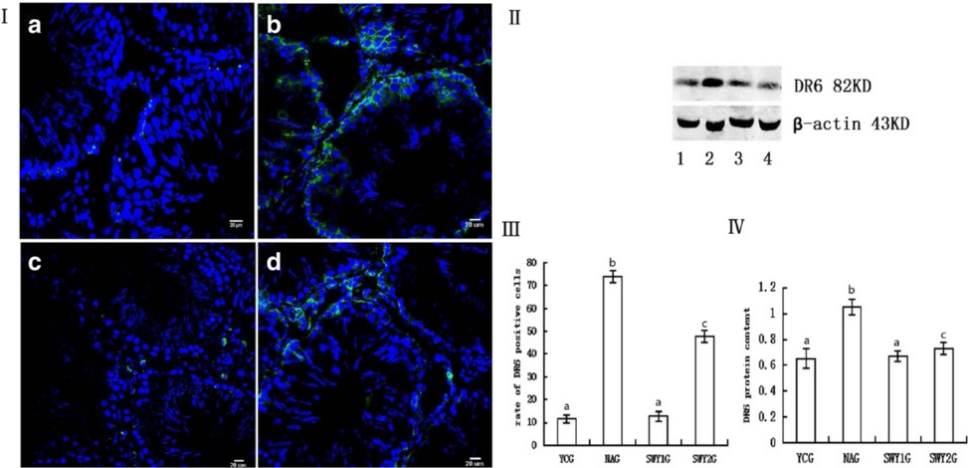
Immunofluorescence and western blot for DR6 and statistical analysis. Immunofluorescence staining was used to detect the expression of DR6 and the rates of positive cells were quantified(I,III). The results showed that the rates of cells positive for DR6 were significantly higher in the NAG than in the YCG (*P* < 0.01). In the SWY1G and SWY2G, the rate of cells positive for DR6 was significantly lower than in the NAG (*P* < 0.01). **a**: young control group (YCG); **b**: natural aging group (NAG); **c**: Heshouwuyin group 1 (SWY1G); **d**: Heshouwuyin group 2 (SWY2G). Western blot was used to detect the content of DR6 protein and statistical analysis was performed according to the gray level(II,IV). The results showed the variation trend was consistent with the results of immunofluorescence.1: young control group (YCG); 2: natural aging group (NAG); 3: Heshouwuyin group 1 (SWY1G); 4: Heshouwuyin group 2 (SWY2G). Each histogram represents the mean ± SD (*n* = 3). Columns with different letters represent significantly different values, while the same letters indicate no significant difference. Bars = 20 μm

Immunofluorescence staining showed that the positive products of DR6 were green granules distributed throughout the cytoplasm. In the YCG, DR6 protein was mainly located in the spermatogonia; in the NAG, SWY1G and SWY2G, DR6 protein was mainly located in spermatogonia and a few Leydig cells. The rate of positive cells was significantly higher in the NAG than in the YCG (*P* < 0.01) and significantly lower in the SWY1G, SWY2G than in the NAG (*P* < 0.01), the rate of positive cells in the SWY1G was significantly lower than in the SWY2G (*P* < 0.01). Western blot was used to detect the content of DR6 in the testes. Semi-quantitative statistical analysis showed that the variation trend was consistent with the results of immunofluorescence (Fig. 10).

### Expression of Cytc protein

**Fig. 11:**
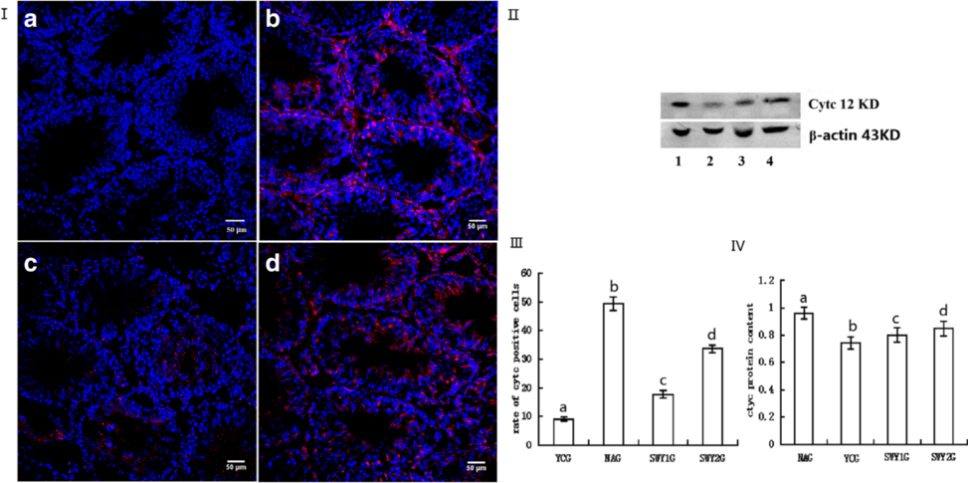
Immunofluorescence and western blot for Cytc and statistical analysis. Immunofluorescence staining was used to detect the expression of Cytc and the rates of positive cells were quantified(I,III). The results showed that the rates of cells positive for Cytc were significantly higher in the NAG than in the YCG (*P* < 0.01). In the SWY1G and SWY2G, the rate of cells positive for Cytc was significantly lower than in the NAG (*P* < 0.01). **a**: young control group (YCG); **b**: natural aging group (NAG); **c**: Heshouwuyin group 1 (SWY1G); **d**: Heshouwuyin group 2 (SWY2G). Western blot was used to detect the content of Cytc protein and statistical analysis was performed according to the gray level(II,IV). The results showed the variation trend was consistent with the results of immunofluorescence.1: natural aging group (NAG); 2: young control group (YCG); 3: Heshouwuyin group 1 (SWY1G); 4: Heshouwuyin group 2 (SWY2G). Each histogram represents the mean ± SD (*n* = 3). Columns with different letters represent significantly different values, while the same letters indicate no significant difference. Bars = 50 μm

Immunofluorescence staining showed that the positive products of Cytc were red granules distributed throughout the cytoplasm. In the YCG, Cytc protein was mainly located in Leydig cells; in the NAG and SWY2G, Cytc protein was mainly located in spermatogonia, spermatocytes, Leydig cells, and a few sperm cells; and in the SWY1G, Cytc protein was mainly located in spermatogonia and spermatocytes. The rate of positive cells was significantly higher in the NAG than in the YCG (*P* < 0.01) and significantly lower in the SWY1G, SWY2G than in the NAG (*P* < 0.01), the rate of positive cells in the SWY1G was significantly lower than in the SWY2G (*P* < 0.01). Western blot was used to detect the content of Cytc in the testes. Semi-quantitative statistical analysis showed that the variation trend was consistent with the results of immunofluorescence (Fig. 11).

### Expression of 14-3-3σ protein

**Fig. 12:**
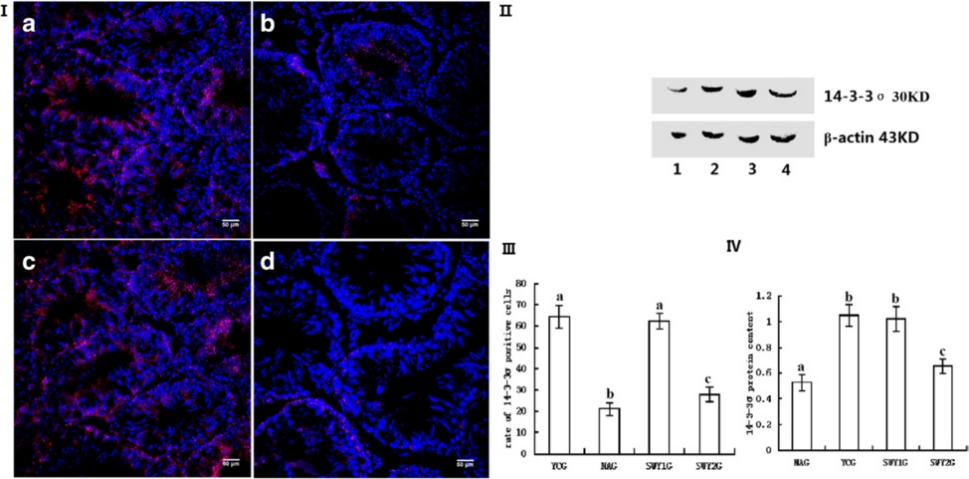
Immunofluorescence and western blot for 14-3-3σ and statistical analysis. Immunofluorescence staining was used to detect the expression of 14-3-3σ and the rates of positive cells were quantified(I,III). The results showed that the rates of cells positive for 14-3-3σ were significantly lower in the NAG than in the YCG (*P* < 0.01). In the SWY1G and SWY2G, the rate of positive cells was significantly higher than in the NAG (*P* < 0.01). **a**: young control group (YCG); **b**: natural aging group (NAG); **c**: Heshouwuyin group 1 (SWY1G); **d**: Heshouwuyin group 2 (SWY2G). Western blot was used to detect the content of 14-3-3σ protein and statistical analysis was performed according to the gray level(II,IV). The results showed the variation trend was consistent with the results of immunofluorescence.1: natural aging group (NAG); 2: young control group (YCG); 3: Heshouwuyin group 1 (SWY1G); 4: Heshouwuyin group 2 (SWY2G). Each histogram represents the mean ± SD (n = 3). Columns with different letters represent significantly different values, while the same letters indicate no significant difference. Bars = 50 μm

Immunofluorescence staining showed that the positive products of 14-3-3σ were red granules distributed throughout the cytoplasm. In the YCG and SWY1G, 14-3-3σ protein was mainly located in spermatogonia, spermatocytes, and sperm cells; in the NAG and SWY2G, 14-3-3σ protein was mainly located in Leydig cells and a few spermatogonia. The rate of positive cells was significantly lower in the NAG than YCG (*P* < 0.01) and significantly higher in the SWY1G, SWY2G than in the NAG (*P* < 0.01), the rate of positive cells in the SWY1G was significantly higher than in the SWY2G (*P* < 0.01). Western blot was used to detect the content of 14-3-3σ in the testes. Semi-quantitative statistical analysis showed that the variation trend was consistent with the results of immunofluorescence (Fig. 12).

## Discussion

Traditional Chinese medicine helps to delay aging by increasing the antioxidant capacity, enhancing immunity, and regulating the blood lipid concentrations [8, 9]. Additionally, it have produced good clinical efficacy, with little toxicity and few side effects. Because the mechanism is still unclear, experimental datas were yet to be consummated. In the present study, Wistar rats were used as an animal model of natural aging. Although these rats have relatively high mortality during the long breeding cycle, they effectively reproduced the aging process in humans. Recent studies have confirmed that a variety of single herbs and Chinese medicines have anti-aging and anti-apoptotic effects. Yuan et al. showed that *Epimedium* flavonoids can inhibit cyclophosphamide-induced apoptosis of spermatogenic cells. *Polygonum multiflorum* has been shown to inhibit apoptosis of human umbilical vein endothelial cells and down-regulate the expression of caspase-3 [10]. Salvianolic acids, the water-soluble ingredient of *Salvia miltiorrhiza*, were shown to have a protective effect on TNF-α/D-galactosamine-treated hepatocyte LO2 cells. Salvianolic acids were also shown to antagonize endoplasmic reticulum stress and mitochondrial-dependent apoptosis induced by TNF-α/D-amino galactose by modulating the Bax/Bcl-2 ratio and calcium release [11]. Heshouwuyin, a kind of Chinese herb used to tonify the kidney, comprises Heshouwu pill, *Herba epimedii*, *Salvia miltiorrhiza*, and *Poria*. Heshouwu pills are a traditional clinical prescription in the book titled *Xuan Ming Lun*, written by Hejian Liu. They are based on *Polygonum multiflorum* and supplemented with *cistanche* and *achyranthes root*, and have exhibited reliable long-term clinical efficacy. *Herba epimedii* can tonify the kidney to strengthen muscles and bones, *Salvia miltiorrhiza* can promote circulation and remove stasis to dredge angiocarpy, and *Poria* can invigorate the spleen and eliminate damp to increase mental tranquility. Heshouwuyin significantly alleviates apoptosis of hippocampal neurons induced by β-amyloid protein and inhibit the apoptosis of ovary cells [12, 13].

This study screened out 21,260 genes by gene microarray analysis and found that 2100 genes were differentially expressed between the YCG and NAG, 1948 genes were differentially expressed between the SWY1G and NAG, and 912 genes were directly regulated by Heshouwuyin. Of the latter 912 genes, 691 were significantly up-regulated in the NAG and 221 were significantly down-regulated compared with the YCG. After Heshouwuyin intervention, the 691 genes were significantly down-regulated, the 221 genes were significantly up-regulated, and 65 of the 912 genes were related to cell apoptosis. These genes are involved in many biological processes such as proliferation, immunity, metabolism, cell growth, and cell death. They are mainly distributed in 59 signal pathways, indicating that Heshouwuyin can regulate the process of testis senescence in a variety of ways.

Modern studies have demonstrated that apoptosis plays an important role in clearing abnormal sperm cells and adjusting the quantity and quality of sperm [14]. Along with the increase in the rate of testicular germ cell apoptosis because of aging, the number of abnormal sperm increased significantly [15] and spermatogenic arrest was usually found in the primary spermatocyte [16] or sperm cell stage; the most obviously decreased was spherical sperm or sperm cells [17]. Syed and Hecht [18] pointed out that the loss of ability to induce Sertoli cells in aged mice was also an important factor which leading to sperm apoptosis. In the present study, TUNEL staining and flow cytometry results showed that the rate of testicular cell apoptosis in the naturally aging rats was significantly increased. Additionally, cell cycle detection revealed that the spermatogenic cells of naturally aging rats exhibited obvious G1 phase arrest and the number of cells in the S and G2 phases were decreased. All of these findings indicate that the proliferation ability of spermatogenic cells in naturally aging rats was significantly decreased, which is consistent with previous research. After the Heshouwuyin intervention, the rate of testicular cell apoptosis in natural aging rats was significantly decreased and the cell proliferation index was improved. This indicates that Heshouwuyin can inhibit apoptosis of testicular cells and promote cell proliferation in aging rats; however, the mechanism of this effect remains unclear.

The mitochondrial apoptosis pathway plays an important role in the regulation of spermatogenic cell apoptosis. When cells are impacted by internal apoptosis-stimulating factors such as DNA damage and hypoxia, the mitochondrial apoptosis pathway may be activated, resulting in the induction of cell apoptosis. In this way, the apoptosis signal either directly or indirectly (through the Bcl-2 family) induces the increase in mitochondrial membrane permeability [19], and leading to Cytc releasing into the cytoplasm where Cytc binds to Apaf-1 and procaspase-9 [20], thereby activating caspase and inducing cell apoptosis. BAX and Bad are important pro-apoptotic factors of the Bcl-2 family, and they are distributed in the cytoplasm of spermatogenic cells. Once induced, they move toward the cell nucleus and locate in the mitochondrial outer membrane next to the nucleus to form multimers, thus promoting the release of Cytc into the cytoplasm. Active downstream caspase-3 (activation or expression of caspase-3 can be used as a marker of cell apoptosis) initiates the caspase cascade and induces apoptosis. Meanwhile, Cytc accumulates in the cytoplasm, resulting in a reduction of Cytc oxidase transported by Cytc. Electrons are then released from the respiratory chain and form superoxide anion in combination with oxygen. The superoxide anion turns into reactive oxygen molecules and hydrogen peroxide; however, the two substances are potentially toxic and induce cell apoptosis. High concentrations of BAX can initiate cell death, while low concentrations can make organelles release active molecules that activate caspases and antagonize Bcl-2, resulting in cell apoptosis [21]. BAX is a key factor in the upstream mitochondrial apoptosis pathway. Many apoptotic factors, including DR6 and IGFBP3, induce their effect through interaction with BAX [22–24]. 14-3-3σ protein can inhibit apoptosis induced by Bad mainly via blocking the interaction between Bad and Bcl-xl or Bcl-2, then Bad is found in the cytosol, bound to 14–3-3 proteins, and this form of Bad does not promote apoptosis [25]. Many studies have shown that caspase-3, BAX, DR6, 14-3-3σ and Cytc were key factors in the mitochondrial apoptotic pathway [26, 27]. In this study, qRT-PCR was used to detect changes in mRNA levels of these genes. The results showed that expression of caspase-3, BAX, DR6, SOCS3, GSY2 and Cytc was significantly up-regulated and that the expression of 14-3-3σ was significantly down-regulated in natural aging rats’ testes; the Heshouwuyin intervention reversed these phenomena. Immunofluorescence and western blot analysis were used to detect the changes in caspase-3, BAX, DR6 and 14-3-3σ protein levels, and the results were consistent with mRNA levels.

Because Heshouwuyin is composed of many kinds of traditional Chinese medicine, the chemical components of Heshouwuyin are not very clear. According to the predecessors’ research, we found that both of the 2, 3, 5, 4’ -tetrahydroxystilbene-2-O-β-D-gluco-side of *Polygonum multiflorum*, Echinacoside glycosides of *Herba Cistanche*, Icariin of *Herba epimedii* and Tanshinone IIA of *Salvia miltiorrhiza* could inhibit cell apoptosis by regulating the key gene expression through mitochondrial pathway [28–31]. In addition, the prepared *Polygonum multiflorum* is chosen in Heshouwuyin which has little toxicity, it is compatible with the other kinds of medicine and these medicines reinforce each other which ensures Heshouwuyin exact efficacy. Therefore, besides the proposed components, maybe there other effective chemical components, and the exactly components need to be further studied.

## Conclusions

Our findings suggest that the effect of Heshouwuyin on inhibiting apoptosis of spermatogenic cells may be associated with the mitochondrial apoptosis pathway. Heshouwuyin reduces the release of Cytc by up-regulating the anti-apoptotic factor 14-3-3σ, down-regulating DR6 and BAX, and preventing interaction of Bad with Bcl-xl or Bcl-2, thus inhibits the mitochondrial apoptosis pathway and reduces apoptosis of spermatogenic cells. Heshouwuyin therefore plays a role in anti-apoptosis, although the mechanisms need to be further studied.

## Abbreviations

PBS: Phosphate-buffered saline
DAPI: 4′,6-diamidino-2-phenylindole
DR6: Death receptor 6
Caspase: Cysteinyl aspartate specific proteinase
Cytc: Cytochrome c
